# Improvement in Quality of Life After Early Interactive Human Coaching via a Mobile App in Postgastrectomy Patients With Gastric Cancer: Prospective Randomized Controlled Trial

**DOI:** 10.2196/75445

**Published:** 2025-12-18

**Authors:** Bang Wool Eom, Mira Han, Hong Man Yoon, Young-Woo Kim, So Young Kim, Jin Myoung Oh, Gyung Ah Wie, Keun Won Ryu

**Affiliations:** 1 Center for Gastric Cancer National Cancer Center Goyang Republic of Korea; 2 Department of Medical Research Collaborating Center Seoul Metropolitan Government -Seoul National University Boramae Medical Center Seoul Republic of Korea; 3 Department of Clinical Nutrition National Cancer Center Goyang Republic of Korea

**Keywords:** human coaching, mobile, gastric cancer, gastrectomy, quality of life

## Abstract

**Background:**

Patients undergoing gastrectomy usually experience postgastrectomy syndrome and face difficulties adapting to a regular diet. Human health coaching via a mobile app has recently been applied to patients with chronic metabolic diseases, with significant improvements being observed in clinical outcomes.

**Objective:**

This study aimed to compare the quality of life and nutritional outcomes of human health coaching via a mobile app with those of conventional face-to-face counseling in postgastrectomy patients with gastric cancer.

**Methods:**

This was a prospective randomized controlled trial, and patients were enrolled between May 2020 and August 2022. The mobile coaching group received health coaching that provides personalized advice based on self-recorded health data via a mobile app from assigned coaches for 3 months after discharge, and the conventional counseling group underwent dietary consultations with a clinical dietitian 1 and 3 months postoperatively. The primary end point for sample size calculation was the eating restriction score on the European Organisation for Research and Treatment of Cancer Quality of Life Questionnaire gastric cancer module 1 month postoperatively. Secondary end points included changes in other subscales of the European Organisation for Research and Treatment of Cancer Quality of Life Questionnaire Core 30 and gastric cancer module, as well as nutritional outcomes assessed preoperatively and 1, 3, 6, and 12 months postoperatively.

**Results:**

Data from 88.9% (160/180) of enrolled patients were analyzed after excluding dropouts. In the mobile coaching group (n=76), 66% (n=50) of patients who used the mobile app for ≥8 weeks were classified as active users. No significant difference in eating restriction 1 month postoperatively was found between the mobile coaching and conventional counseling groups. However, the mobile coaching group reported less dyspnea during the entire period (*P*=.01), less eating restriction at 6 months (*P*=.045), and less negative body image 3 months postoperatively (*P*=.04) than the conventional counseling group (n=84). Exploratory subgroup analyses based on age, sex, and operation type indicated that younger patients (<60 years), female patients, and those who underwent distal gastrectomy had better quality of life from mobile coaching. In the mobile coaching group, exploratory subgroup analyses based on mobile activity showed that active users had a better global health status than inactive users (*P*=.005). However, no significant differences in body composition or nutritional parameters were observed between the mobile coaching and conventional counseling groups or between active and inactive users in the mobile coaching group.

**Conclusions:**

Although this trial did not show a significant difference in eating restriction 1 month postoperatively, human coaching via a mobile app was associated with fewer symptoms in some scales compared to conventional counseling in postgastrectomy patients with gastric cancer. The intervention might help patients manage their symptoms and adapt to their diet.

**Trial Registration:**

ClinicalTrials.gov NCT04394585; https://clinicaltrials.gov/study/NCT04394585

## Introduction

Radical gastrectomy with lymph node dissection is the standard treatment for localized gastric cancer [1]. This procedure offers curative potential and achieves the most desirable outcome. Despite this benefit, patients usually undergo significant lifestyle changes after gastrectomy, with many experiencing postgastrectomy syndrome—a condition characterized by early satiety, abdominal fullness, indigestion, and dumping syndrome. Therefore, eating and postgastrectomy syndrome management are key aspects of postoperative care for these patients.

Patients receive in-hospital education on dietary progression, starting from sips of water to a soft-blend diet, before encountering these challenges. The most effective strategy for preventing postgastrectomy symptoms is to consume small amounts several times daily. Patients are encouraged to practice eating slowly and gradually increasing the amount of food. However, no detailed guidelines exist for transitioning from a soft-blend diet to a regular diet after discharge. Given that each patient has different eating habits, food preferences, digestive abilities, and home environments, applying a uniform guideline is inappropriate. Consequently, dietary adaptation must be tailored to each patient’s situation. Personalized coaching and education may help patients navigate this transition more effectively and reduce the burden of trial and error.

Human health coaching is a patient-centered educational intervention that uses a relational approach to promote sensemaking in health information and support goal setting for health behavior changes [2]. Interpersonal relationships have been shown to play a significant role in achieving specific goals by enhancing self-esteem and self-control [3-5]. Human health coaching has been proven effective in various chronic diseases. However, only a few studies have evaluated its impact in postgastrectomy patients [6].

Therefore, this study aimed to compare the quality of life (QoL) and nutritional outcomes of postgastrectomy patients with gastric cancer undergoing human health coaching via a mobile app with those of patients undergoing conventional face-to-face counseling. Regarding QoL, the primary end point, we hypothesized that the mobile coaching group would experience fewer eating restriction symptoms than the conventional counseling group 1 month postoperatively, which formed the basis for the sample size calculation.

## Methods

### Study Design and Patients

This investigator-initiated prospective randomized controlled trial was conducted at the National Cancer Center in the Republic of Korea. Patients who met the following inclusion criteria were recruited for this study: (1) age ≥19 years, (2) diagnosis of clinical stage 1 gastric cancer based on preoperative esophagogastroduodenoscopy and computed tomography, (3) being scheduled for distal or total gastrectomy, (4) ability to access a mobile app via a smartphone or residence with a caregiver who can access the mobile app, and (5) cognitive capability to communicate with an assigned coach. The dropout criteria were (1) patients whose planned gastrectomy was not performed, (2) patients who received adjuvant chemotherapy for pathological stage 2 or 3 gastric cancer, (3) patients with a hospital stay of ≥3 weeks due to postoperative complications, and (4) patients who requested to withdraw from the study.

### Ethical Considerations

The institutional review board of the National Cancer Center approved the protocol of this randomized controlled trial (NCC2019-0137). The study was conducted in accordance with the ethical standards of the institutional research committees and the principles of the Declaration of Helsinki. Written informed consent was obtained from all patients before recruitment, and they were informed of their right to withdraw from the study at any time without consequence. No financial compensation was provided for participation. Patients were assigned a study number after registration, and data analyses were performed using an anonymized dataset to ensure participant privacy and confidentiality. This study is registered at ClinicalTrials.gov (NCT04394585).

### Randomization

Enrolled patients were registered at the Clinical Research Coordination Center through a web-based clinical trial management system (myTrial; BethesdaSoft Co, Ltd). Randomization was performed using myTrial, allocating patients to either the mobile coaching or conventional counseling group (parallel) in a 1:1 ratio. The stratification factor was based on the extent of gastrectomy (distal vs total).

### Procedures

Patients underwent curative distal or total gastrectomy with D1+ or D2 lymphadenectomy depending on the tumor location and clinical stage [1]. They received standard postoperative management during their hospital stay.

**Figure 1:**
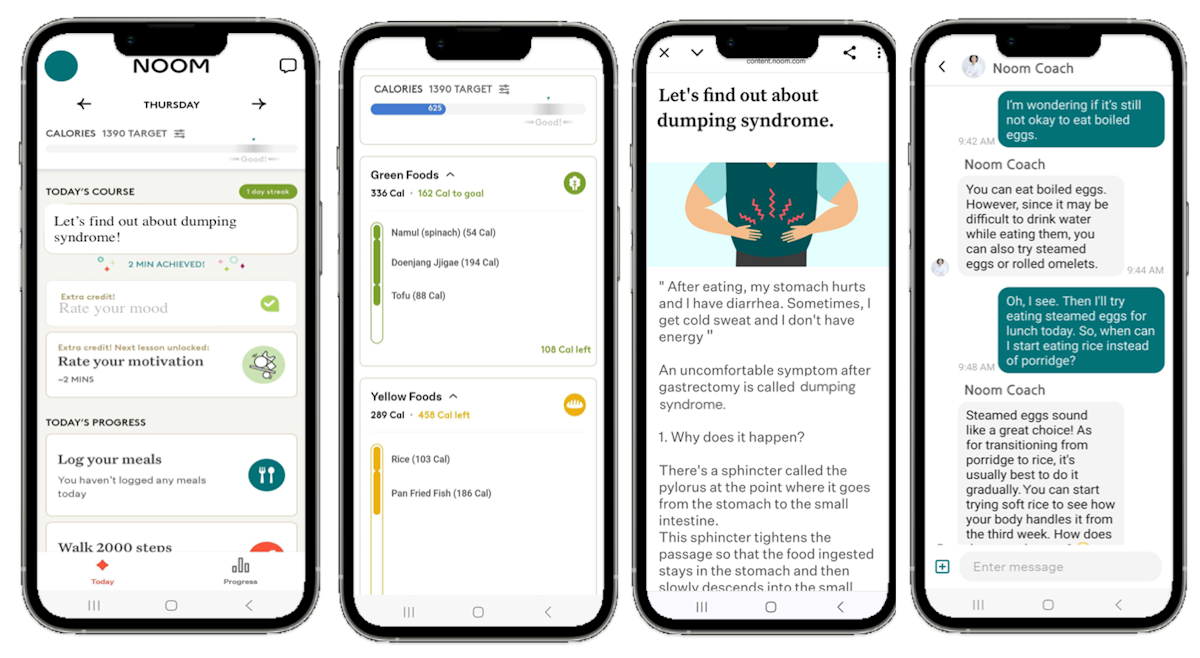
Screenshots of the Noom app for the participants.

Before discharge, patients assigned to the mobile coaching group were instructed to download Noom Coach (Noom Inc) on their smartphones and were guided on how to use it. Thereafter, they received comprehensive human health coaching via the Noom app for 3 months after discharge. Noom Coach is a commercial mobile app that connects a patient with a coach (dietitian) for advice, instruction, and encouragement. Patients were encouraged by a matched coach to log their daily food intake, exercise, and body weight. The app allowed users to record the type and quantity of food consumed, automatically calculating and displaying the total caloric intake (Figure 1).

Exercise was also tracked through manual logging, with patients setting and reporting exercise intensity based on distance and time. The mobile app has a pedometer function that automatically counts the number of steps while it is in use. Informative in-app articles (12 on nutritional or diet-related information, 7 on postgastrectomy syndrome, and 7 on physical activities and mindfulness) were provided 2 or 3 times weekly according to the planned schedule. Coaches monitored patients’ log-in records and whether they had read the articles. They also communicated with patients using an in-app messenger system at least 2 times weekly to encourage them to follow the dietary guidelines, providing individualized feedback based on each patient’s life log. Patients could submit questions related to nutritional and symptomatic issues at any time via the mobile app, and the assigned coach responded as promptly as possible no later than the following morning.

All patients assigned to the conventional counseling group received face-to-face consultations with a hospital-based dietitian 1 and 3 months postoperatively during their routine outpatient visits for regular checkups. During these sessions, patients asked questions regarding their diet, and the dietitian suggested appropriate solutions. Dietitians also provided useful information and brochures when necessary.

### Outcomes

The primary outcome was the eating restriction scale of the European Organisation for Research and Treatment of Cancer Quality of Life Questionnaire (EORTC QLQ) gastric cancer module (EORTC QLQ STO22) 1 month postoperatively. In a previous pilot study involving 20 postgastrectomy patients who used Noom Coach, the eating restriction scale showed the greatest change in score postoperatively, with a significant persisting score difference.

Secondary outcomes were changes in QoL over time as assessed using the EORTC QLQ Core 30 (C30) and EORTC QLQ-STO22. The EORTC QLQ Core 30 (EORTC QLQ-C30) is a 30-item questionnaire that evaluates the general QoL of patients with cancer and comprises 6 functions, 9 symptoms, and global health status. The EORTC QLQ-STO22 is a 22-item, gastric cancer–specific questionnaire comprising 9 symptom scales, each scored from 0 to 100. A higher score on the functional and global health status scales indicates better functional capacity and QoL, whereas a higher score on symptom scales reflects more severe symptoms and poorer QoL [7].

Other secondary outcomes included nutritional parameters such as body weight, BMI, body composition (muscle mass, fat mass, body fat percentage, and basal metabolism), and nutrition-related laboratory values (hemoglobin, serum protein, albumin, and total cholesterol). Body composition was estimated at the outpatient clinic using a bioelectrical impedance analyzer (InBody). The patients completed the questionnaires and assessed their nutritional parameters preoperatively and 1, 3, 6, and 12 months postoperatively.

App activity was also analyzed over 3 months as measured via the patients’ frequency of self-reported food intake and body weight, as well as the number of times they read the articles and messaged their coach. If any data were recorded by the patient in a given week, the mobile app was considered “active” for that week. Patients with app activity for ≥8 and <8 weeks were defined as “active users” and “inactive users,” respectively. The frequency of each app activity was assessed, and the primary and secondary outcomes of the active and inactive user groups were compared.

### Statistical Analyses

The sample size was calculated based on the results of a pilot study [8]. In the pilot study, the eating restriction scale score on the EORTC QLQ-STO22 showed the most significant change—30 points—1 month postoperatively. We assumed the superiority of the mobile coaching group, expecting a 5-point difference with an SD of 10 in the eating restriction scale 1 month postoperatively [8,9]. On the basis of an effect size of 0.5, a type 1 error of 0.05, statistical power of 80%, and a 1:1 allocation ratio, a total of 126 participants were required for analysis using a 2-tailed *t* test. Allowing for a 30% dropout rate, we planned to recruit 180 participants (90 per group).

Normal distribution of continuous variables was assessed using the Shapiro-Wilk test. Descriptive statistics were presented as means and SDs for continuous variables and as frequencies and percentages for categorical variables. Intergroup differences were tested using a *t* test or Wilcoxon rank sum test for continuous variables and the chi-square or Fisher exact test for categorical variables.

Intergroup comparisons were performed using linear mixed models with repeated measures to analyze the changes in QoL and nutritional parameters over time. Fixed effects included groups, time, and the interaction between group and time. Between-group differences in QoL scores and nutritional values were estimated using the least squares means. Post hoc analyses were performed using the Bonferroni correction when the significance of the interaction between group and time was determined. This process identified significant between-group differences at any given time point. Graphs were constructed to illustrate the mean values over time for the QoL subscale scores and nutritional parameters.

The relationship between the mobile app activities (meal input, exercise input, sending messages, reading articles, and body weight input) and outcomes (QoL and nutritional parameters) was assessed using Pearson correlation analyses. We explored which mobile app activities were most closely associated with improvements in QoL and nutritional outcomes.

Data analyses and visualization were conducted using SAS (version 9.4; SAS Institute) and Stata (version 18.0; StataCorp), respectively. Statistical significance was set at *P*<.05.

## Results

### Patients

**Figure 2:**
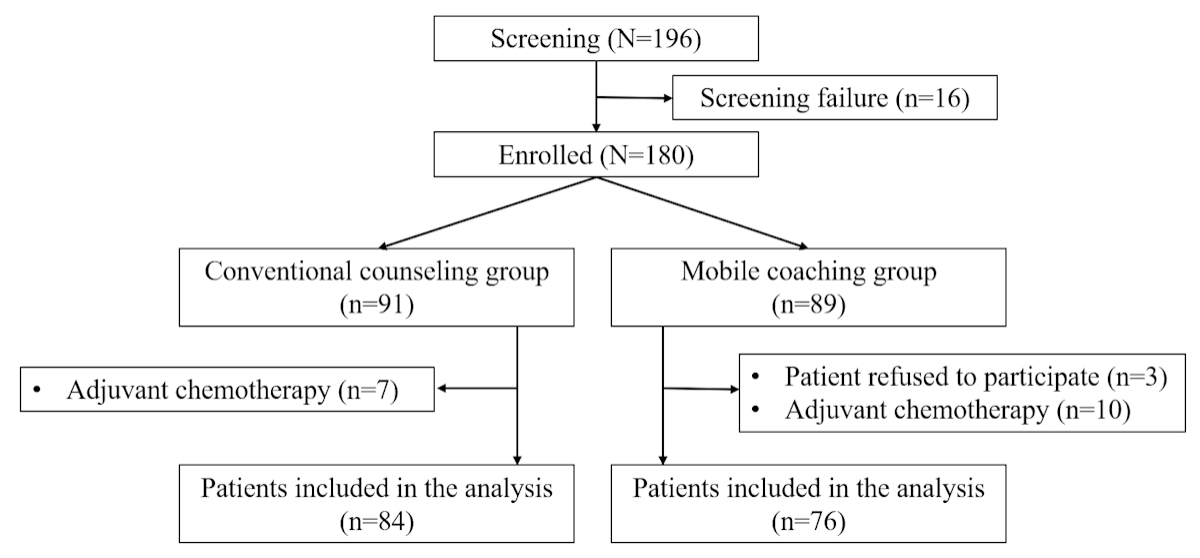
CONSORT (Consolidated Standards of Reporting Trials) diagram.

A total of 196 patients were assessed for eligibility between May 2020 and August 2022. Of these 196 patients, 180 (91.8%) were enrolled in this study and randomly assigned to either the mobile coaching (n=89, 49.4%) or conventional counseling (n=91, 50.6%) group (Figure 2). Twenty patients were excluded because they were receiving adjuvant chemotherapy (n=17, 85%) or withdrew informed consent (n=3, 15%). Ultimately, 160 patients were included in the final analysis.

Patients’ clinicopathological characteristics, including age, sex, BMI, comorbidities, pathological factors, and operation type, did not differ significantly between the conventional counseling and mobile coaching groups (Table 1). The mean ages were 58.7 (SD 9.7) and 60.8 (SD 9.5) years in the conventional counseling and mobile coaching groups, respectively.

**Table 1:** Patient clinicopathological characteristics.

			Conventional counseling group (n=84)		Mobile coaching group (n=76)		*P* value
Age (years), mean (SD)			58.7 (9.7)		60.8 (9.5)		.18
**Sex, n (%)**							.27
	Male	47 (56)		49 (64.5)			
	Female	37 (44)		27 (35.5)			
BMI (kg/m^2^), mean (SD)			23.7 (4.5)		23.9 (3.4)		.73
**Overall comorbidity, n (%)**							.31
	Absent	53 (63.1)		42 (55.3)			
	Present	31 (36.9)		34 (44.7)			
**Detailed comorbidity, n (%)**							
	Hypertension	8 (9.5)		3 (3.9)		.16	
	Hyperlipidemia	13 (15.5)		13 (17.1)		.78	
	Diabetes mellitus	8 (9.5)		17 (22.4)		.03	
	Endocrine disease	4 (4.8)		1 (1.3)		.37^a^	
	Liver disease	4 (4.8)		1 (1.3)		.37^a^	
	Pulmonary disease	1 (1.2)		1 (1.3)		>.99^a^	
	Urinary disease	3 (3.6)		3 (3.9)		>.99^a^	
	Cardiovascular disease	3 (3.6)		8 (10.5)		.08	
	Cerebrovascular disease	2 (2.4)		1 (1.3)		>.99^a^	
	Others	2 (2.4)		1 (1.3)		>.99^a^	
**Location of tumor, n (%)**							.95
	Lower one-third	29 (34.5)		28 (36.8)			
	Middle one-third	46 (54.8)		40 (52.6)			
	Upper one-third	9 (10.7)		8 (10.5)			
**Pathological stage, n (%)**							.48^a^
	IA	67 (79.8)		54 (71.1)			
	IB	16 (19)		21 (27.6)			
	IIIA	1 (1.2)		1 (1.3)			
**Operation type, n (%)**							.95
	Distal	76 (90.5)		69 (90.8)			
	Total	8 (9.5)		7 (9.2)			
**Anastomosis, n (%)**							.95
	Billroth I	8 (9.5)		8 (10.5)			
	Billroth II	68 (81)		60 (78.9)			
	Roux-en-Y	8 (9.5)		8 (10.5)			
Duration of hospital stay (d), median (IQR)			7 (7-7)		7 (6-7)		.91^b^
**Complications, n (%)**							>.99^a^
	Absent	81 (96.4)		73 (96.1)			
	Present	3 (3.6)		3 (3.9)			

^a^Analyzed using the Fisher exact test.

^b^Analyzed using the Wilcoxon rank sum test.

### App Activity in the Mobile Coaching Group

A total of 66% (50/76) and 34% (26/76) of the patients in the mobile coaching group were classified as active and inactive users, respectively (Table 2). There were no significant differences in mean age, sex, mean BMI, comorbidity, pathological stage, operation type, and duration of hospital stay between the 2 groups. Only the complication rate was higher in the inactive user group than in the active user group (3/26, 12% vs 0%; *P*=.04).

**Table 2:** Clinicopathological characteristics of the mobile coaching group.

		Inactive users (n=26)	Active users (n=50)	*P* value	
Age (years), mean (SD)		62.5 (10.6)	59.9 (8.9)	.26	
**Sex, n (%)**					.91
	Male	17 (65.4)	32 (64)		
	Female	9 (34.6)	18 (36)		
BMI (kg/m^2^), mean (SD)		24.3 (4.4)	23.7 (2.9)	.54	
**Overall comorbidity, n (%)**					.51
	Absent	13 (50)	29 (58)		
	Present	13 (50)	21 (42)		
**Detailed comorbidity, n (%)**					
	Hypertension	2 (7.7)	1 (2)	.27^a^	
	Hyperlipidemia	4 (15.4)	9 (18)	>.99^a^	
	Diabetes mellitus	8 (30.8)	9 (18)	.21	
	Endocrine disease	1 (3.8)	0 (0)	.34^a^	
	Liver disease	0 (0)	1 (2)	>.99^a^	
	Pulmonary disease	0 (0)	1 (2)	>.99^a^	
	Urinary disease	3 (11.5)	0 (0)	.04^a^	
	Cardiovascular disease	2 (7.7)	6 (12)	.71^a^	
	Cerebrovascular disease	1 (3.8)	0 (0)	.34^a^	
	Others	1 (3.8)	0 (0)	.34^a^	
**Location of tumor, n (%)**					.84
	Lower	10 (38.5)	18 (36)		
	Middle	14 (53.8)	26 (52)		
	Upper	2 (7.7)	6 (12)		
**Pathological stage, n (%)**					>.99^a^
	IA	19 (73.1)	35 (70)		
	IB	7 (26.9)	14 (28)		
	IIIA	0 (0)	1 (2)		
**Operation type, n (%)**					>.99^a^
	Distal	24 (92.3)	45 (90)		
	Total	2 (7.7)	5 (10)		
**Anastomosis, n (%)**					.68^a^
	Billroth I	4 (15.4)	4 (8)		
	Billroth II	20 (76.9)	40 (80)		
	Roux-en-Y	2 (7.7)	6 (12)		
Duration of hospital stay (days), median (IQR)		7 (7-7)	7 (6-7)	.67^b^	
**Complications, n (%)**					.04^a^
	Absent	23 (88.5)	50 (100)		
	Present	3 (11.5)	0 (0)		

^a^Analyzed using the Fisher exact test.

^b^Analyzed using the Wilcoxon rank sum test.

**Figure 3:**
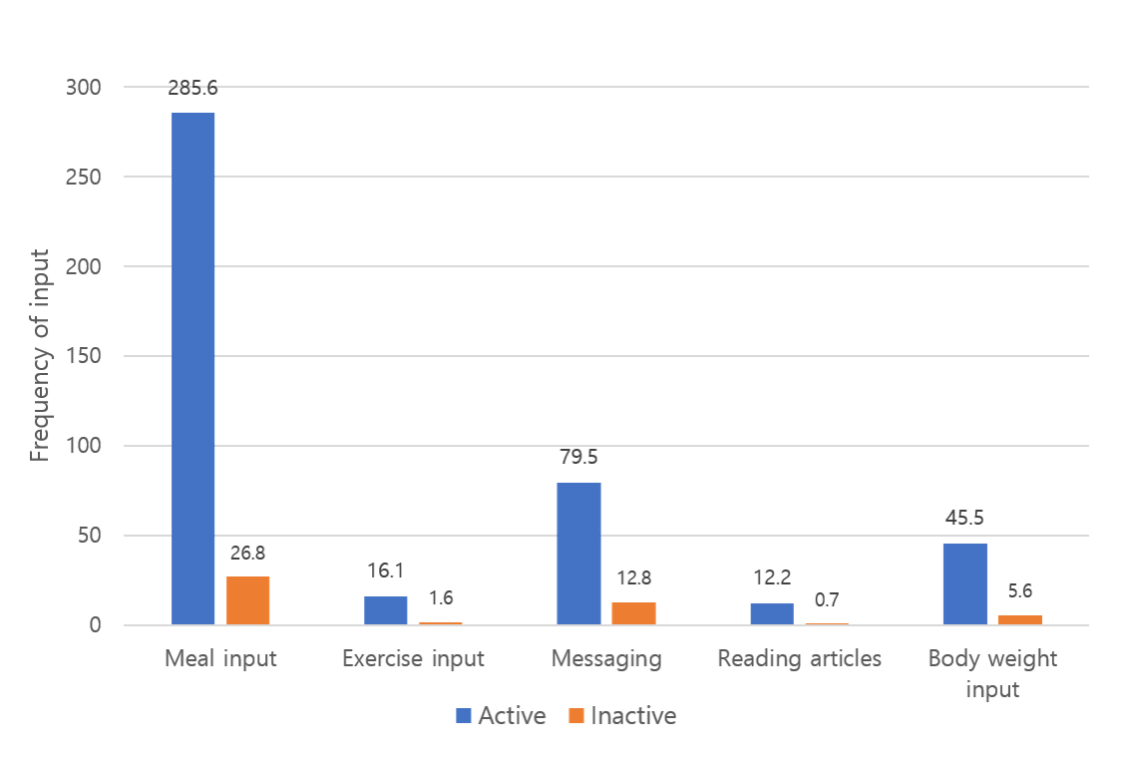
Frequency of input in active and inactive users in the mobile coaching group.

The most frequently performed activities in both groups were meal input, followed by sending messages to coaches, body weight input, exercise input, and reading articles (Figure 3). In the active and inactive user groups, the median number of total activities for 3 months was 487.5 (IQR 234.5-629.3) in the active user group and 16 (IQR 2.8-82.5) in the inactive user group.

**Figure 4:**
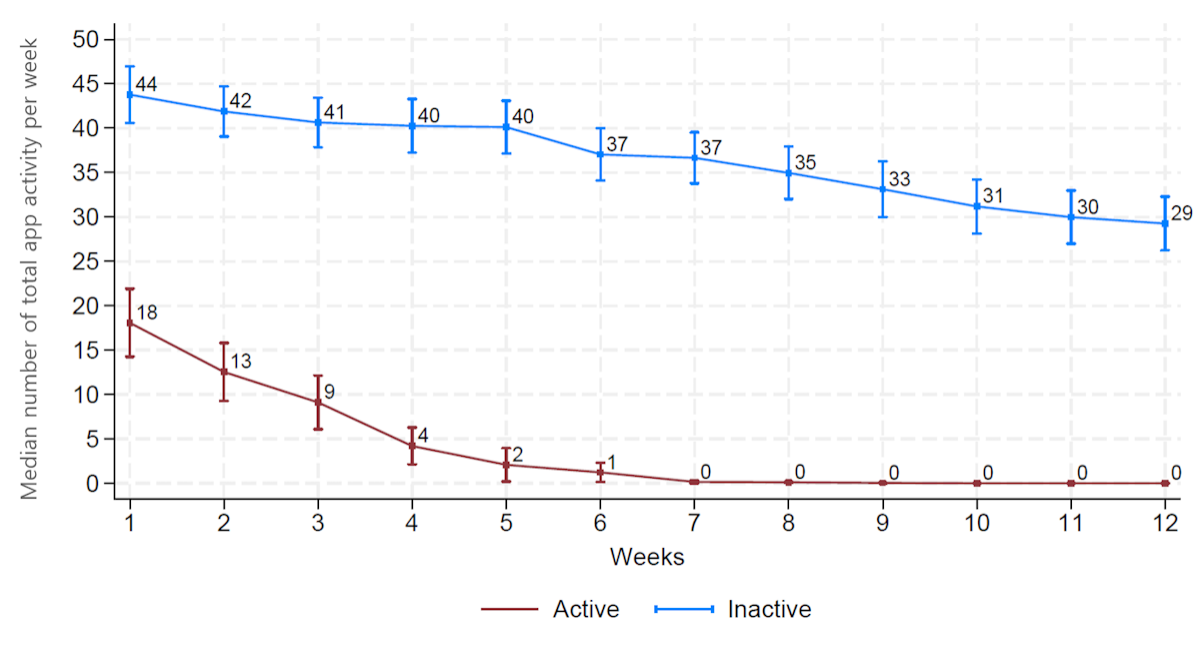
Changes in total app activity per week over time in active and inactive users.

Figure 4 shows the number of total activities per week over time. The active user group had ≥40 log records per week during the first month and maintained ≥30 log records until the last week. In contrast, the inactive user group had <20 log records per week in the first week and no records logged after week 7.

### Eating Restriction Scale and Changes in QoL

The mean eating restriction scale score 1 month postoperatively was 28.2 (SD 19.3) and 27.9 (SD 20.4) in the conventional counseling and mobile coaching groups, respectively (*P*=.89), indicating no significant between-group differences.

**Figure 5:**
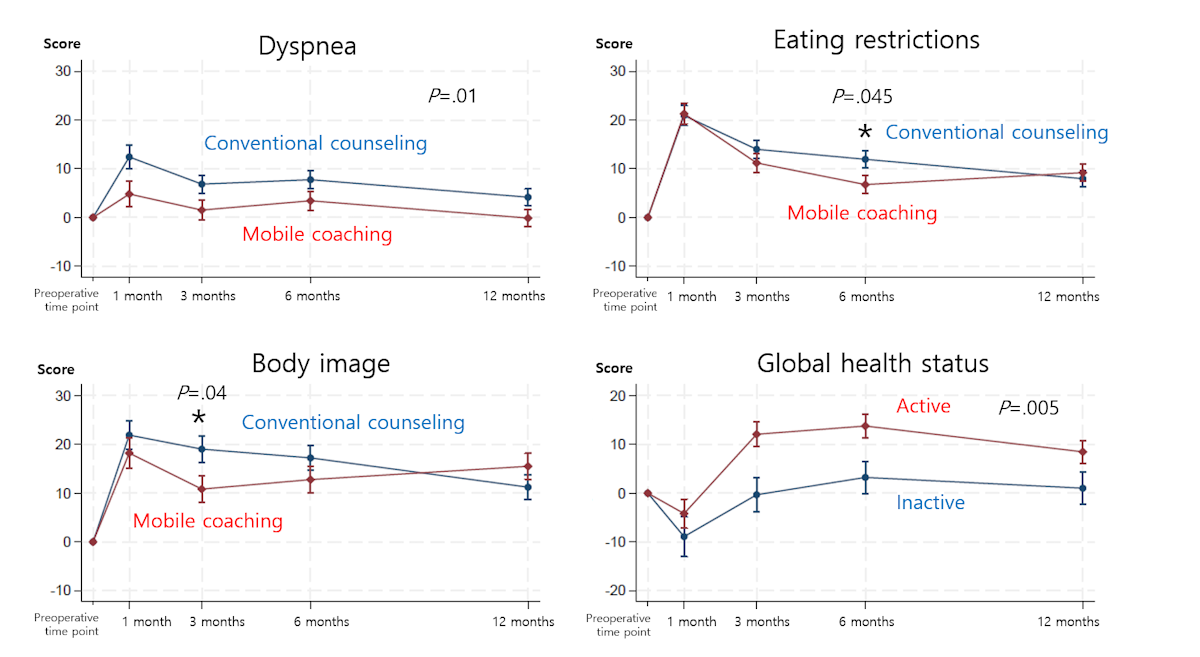
Significant mean changes in postoperative quality of life (QoL).

Figure 5 illustrates the overall changes in postoperative QoL. Compared to the conventional counseling group, the mobile coaching group demonstrated significantly less dyspnea throughout the study period (*P*=.01), reduced eating restriction at 6 months (*P*=.045), and less negative body image 3 months postoperatively (*P*=.04). No significant differences were observed in the remaining functional and symptom scales assessed using the EORTC QLQ-C30 and EORTC QLQ-STO22 (Multimedia Appendices 1-3).

Furthermore, exploratory subgroup analyses based on age, sex, and operation type revealed that the younger age group (<60 years), female individuals, and patients undergoing distal gastrectomy showed a significant association with better QoL from mobile coaching (Tables S1-S3 in Multimedia Appendix 4). The mobile coaching group had less fatigue (*P*=.01) and dyspnea (*P*=.004) and improved body image (*P*=.02) among younger patients; better emotional function (*P*=.03) and less general pain (*P*=.04), dysphagia (*P*=.04), gastric pain (*P*=.04), and dry mouth (*P*=.02) in female individuals; and less fatigue (*P*=.02) and dyspnea (*P*=.001) in patients who underwent distal gastrectomy than the conventional counseling group.

### Changes in Body Composition and Nutritional Parameters

Patients in both groups experienced a postoperative body weight loss of approximately 5 kg, which continued for 1 year postoperatively (Multimedia Appendix 5). No significant differences were observed in any of the body composition parameters, including skeletal muscle mass, body fat mass, body fat percentage, BMI, or basal metabolic rate, between the 2 groups. Similarly, no significant between-group differences were found in changes in nutritional parameters, including hemoglobin, albumin, protein, and total cholesterol levels (Multimedia Appendix 6).

Exploratory subgroup analyses based on age, sex, and operation type also showed no significant benefit from mobile coaching in any of the subgroups (data not shown).

### Outcomes According to App Activity in the Mobile Coaching Group

Exploratory subgroup analyses based on app activity were conducted in the mobile coaching group. The active user group had a significantly better global health status than the inactive user group (*P*=.005; Figure 4). No significant between-group differences were noted in the other functional and symptom scales (Multimedia Appendices 7-9).

Correlations between app activities and changes in QoL were also assessed to identify which activities contributed most to outcomes (Tables S1 and S2 in Multimedia Appendix 10). Weekly engagement with each activity was correlated with QoL subscales, yielding correlation coefficients (*r*) ranging from −0.3 to 0.4. The activity of sending messages exhibited a relatively higher positive correlation with global health status (*r*=0.366), followed by meal input (*r*=0.292) and reading articles (*r*=0.2). Exercise and weight inputs showed very weak correlations with global health status.

## Discussion

### Principal Findings

This study represents the first prospective randomized controlled trial to compare the efficacy of mobile health coaching with that of conventional face-to-face counseling in postgastrectomy patients with gastric cancer. Adherence to the program was considerably high, with approximately two-thirds of the patients (50/76, 66%) actively using the mobile health coaching for 3 months; meal logs were the most frequently entered data. While mobile coaching did not affect patients’ body composition or nutritional parameters, patients in the mobile coaching group reported fewer symptoms, including dyspnea, eating restriction, and negative body image, than the conventional counseling group. In the mobile coaching group, active users were associated with a better global health status compared to inactive users.

### Developing Mobile Coaching for Postgastrectomy Patients

Nutritional and lifestyle interventions via mobile app platforms have been used to manage various chronic metabolic diseases, such as obesity, hypertension, dyslipidemia, and diabetes. Previous studies have reported significant reductions in body weight, BMI, systolic blood pressure, fasting glucose level, and glycated hemoglobin level in participants who used mobile phone apps [10-13]. The use of mobile platforms has expanded to survivors of cancer, particularly those with obesity-associated cancers such as breast, prostate, and colon cancer [14,15]. Postdiagnosis weight gain is common in patients with breast cancer and has been associated with increased risk of recurrence and mortality [16,17]. Therefore, mobile coaching programs have been applied to support patients with breast cancer and have proven to be useful for promoting weight control and improving QoL [18,19].

Postoperative weight maintenance, rather than weight loss, is required in survivors of gastrointestinal cancer. Most patients experience anorexia, dyspepsia, and weight loss after gastric cancer surgery, and malnutrition has been associated with a poor prognosis [20,21]. Therefore, we developed a different mobile coaching app for postgastrectomy patients and trained dieticians on how to respond to the patients’ symptoms and questions [8]. We ensured that the dietitians’ recommendations did not conflict with the surgeons’ advice by asking all surgeons for their opinions on food restriction and any precautions during the dietary adaptation period.

### Adherence to the Mobile App

The applicability of the mobile app in postgastrectomy patients with gastric cancer was an important finding of this study. In previous studies, app activity was evaluated using various parameters, including the mean number of log-ins, median number of days visiting the app, and average time spent on the app, which made it difficult to summarize the results [14]. A recent study on patients with breast cancer revealed that the mobile app was opened a mean of 150 times during a 26-week study period [17]. Another study on weight control in individuals with colorectal polyps showed that 44% of the participants were adherent, which is defined as using a device for more than two-thirds of the follow-up period [22]. In other studies, approximately 59% of patients with pancreatic cancer had app activity for ≥9/12 weeks of follow-up, and 69.4% of patients scheduled for neoadjuvant chemoradiotherapy for esophageal cancer used the app for >6/8 weeks [23,24]. Our study demonstrated similar app activity, with 66% (50/76) of patients whose mean age was 60.8 (SD 9.5) years using the mobile app coaching program for ≥8/12 weeks. These app activity data provide a solid foundation for planning further studies, and adherence could increase over time as people become more familiar with mobile apps.

### Clinical Effectiveness of Mobile Coaching

The mobile coaching program exhibited some favorable results in patient-reported outcomes in this study. Previous studies on survivors of cancer have shown inconsistent patient-reported outcomes and substantial variability attributed to differences in study population, sample size, intervention duration, protocol, and type of comparison group [14]. The aforementioned study on patients with pancreatic cancer found no significant difference in the mean EORTC QLQ-C30 score but reported a significantly better global health status in mobile app users than in nonusers [23]. In our study, scores on the 3 symptom scales of the EORTC QLQ were significantly lower in the mobile app group than in the conventional counseling group. The advice and support provided by the coaches may have fostered a greater sense of comfort and confidence in patients. This, in turn, may have contributed to the reduction in dyspnea and eating restrictions, as well as helping patients have a positive body image.

However, the mobile coaching program did not affect nutritional outcomes in this study. No differences were observed in any of the nutritional parameters between the mobile coaching and conventional counseling groups or between active and inactive users in the mobile coaching group. The previous study on patients with pancreatic cancer also demonstrated no differences in body weight or BMI between the 2 groups [23]. However, the skeletal muscle mass index was maintained in active users but decreased considerably in non–app users after 2 months. This difference may have been related to the fact that active users input exercise and recorded step counts more frequently than inactive users. In our study, patients received mobile coaching immediately after discharge from gastrectomy, a period when most of them experienced eating difficulty and general weakness. Therefore, patients are likely to be primarily concerned with eating challenges and may not have been motivated to engage in exercise. The intensity and duration of coaches’ interventions may also have been insufficient to have a substantial impact on objective nutritional or body composition outcomes. Thus, exercise input was the least frequent app activity, with only 16 exercise activities recorded even among active users over 3 months. In the future, more proactive and timelier patient-tailored approaches are required to help patients maintain their muscle mass and encourage regular exercise.

### Limitations

This study had some limitations. First, the hypothesis for calculating sample size was not met, and the actual statistical power appeared to be lower than expected. Although the primary hypothesis (reduction in eating restriction 1 month postoperatively) was not proven, other scales showed significant differences between the 2 groups. These findings will help formulate a more appropriate hypothesis for a future study. Second, this trial was not designed or powered to assess significance within subgroups, and there is a possible selection bias due to covariate imbalance. Therefore, results from subgroup analyses require careful interpretation. Third, the mobile coaching program was available only for 3 months, and its long-term effects were not studied. If we are more proactive in coaching exercises over the long term, this might result in differences in body composition and additional QoL scales. Fourth, although this study had no age limit, some older patients or patients who had difficulty using smartphones were excluded. Moreover, patients were recruited from a single institution in South Korea, limiting the applicability of these study results to other populations worldwide. Finally, as this trial was not a blinded study, an expectation or reporting bias could have been generated.

### Conclusions

This study found that 66% (50/76) of patients who underwent gastrectomy (mean age 60.8, SD 9.5 years) demonstrated good adherence to a mobile coaching program designed for dietary adaptation. A 3-month human health coaching delivered via a mobile app was associated with a reduction in symptoms across several scales when compared to conventional face-to-face counseling. This program has the potential to assist patients in managing their symptoms and facilitating adaptation to their regular diet. Furthermore, with modification, it may be applicable to supporting patients with other cancer types who require dietary or lifestyle coaching.

## Data Availability

The datasets generated or analyzed during this study are available from the corresponding author on reasonable request.

## Appendix

Multimedia Appendix 1 Mean changes in global health status and functioning scale scores assessed using the European Organisation for Research and Treatment of Cancer Quality of Life Questionnaire Core 30 between the mobile coaching and conventional counseling groups.

Multimedia Appendix 2 Mean changes in general symptom scale scores assessed using the European Organisation for Research and Treatment of Cancer Quality of Life Questionnaire Core 30 between the mobile coaching and conventional counseling groups.

Multimedia Appendix 3 Mean changes in gastric cancer–specific symptom scale scores assessed using the European Organisation for Research and Treatment of Cancer Quality of Life Questionnaire gastric cancer module between the mobile coaching and conventional counseling groups.

Multimedia Appendix 4 Subgroup analyses based on clinical factors for significance of differences in postoperative quality of life between the mobile coaching and conventional counselling groups.

Multimedia Appendix 5 Mean changes in body weight and body composition between the mobile coaching and conventional counseling groups.

Multimedia Appendix 6 Mean changes in nutritional parameters between the mobile coaching and conventional counseling groups.

Multimedia Appendix 7 Mean changes in global health status and functioning scale scores assessed using the European Organisation for Research and Treatment of Cancer Quality of Life Questionnaire Core 30 between the active and inactive user groups.

Multimedia Appendix 8 Mean changes in general symptom scale scores assessed using the European Organisation for Research and Treatment of Cancer Quality of Life Questionnaire Core 30 between the active and inactive user groups.

Multimedia Appendix 9 Mean changes in gastric cancer–specific symptom scale scores assessed using the European Organisation for Research and Treatment of Cancer Quality of Life Questionnaire gastric cancer module between the active and inactive user groups.

Multimedia Appendix 10 Correlations between app activity and quality of life score.

Multimedia Appendix 11 CONSORT-EHEALTH (Consolidated Standards of Reporting Trials of Electronic and Mobile Health Applications and Online Telehealth; version 1.6) checklist.

## Abbreviations

EORTC QLQ: European Organisation for Research and Treatment of Cancer Quality of Life Questionnaire
EORTC QLQ-C30: European Organisation for Research and Treatment of Cancer Quality of Life Questionnaire Core 30
EORTC QLQ-STO22: European Organisation for Research and Treatment of Cancer Quality of Life Questionnaire gastric cancer module
QoL: quality of life
