# Supplementary figures and images for: Improvement in Quality of Life After Early Interactive Human Coaching via a Mobile App in Postgastrectomy Patients With Gastric Cancer: Prospective Randomized Controlled Trial
Source: JMIR Mhealth Uhealth. 2025 Dec 18;13:e75445. doi: 10.2196/75445 (PMC12757711; doi:10.2196/75445)

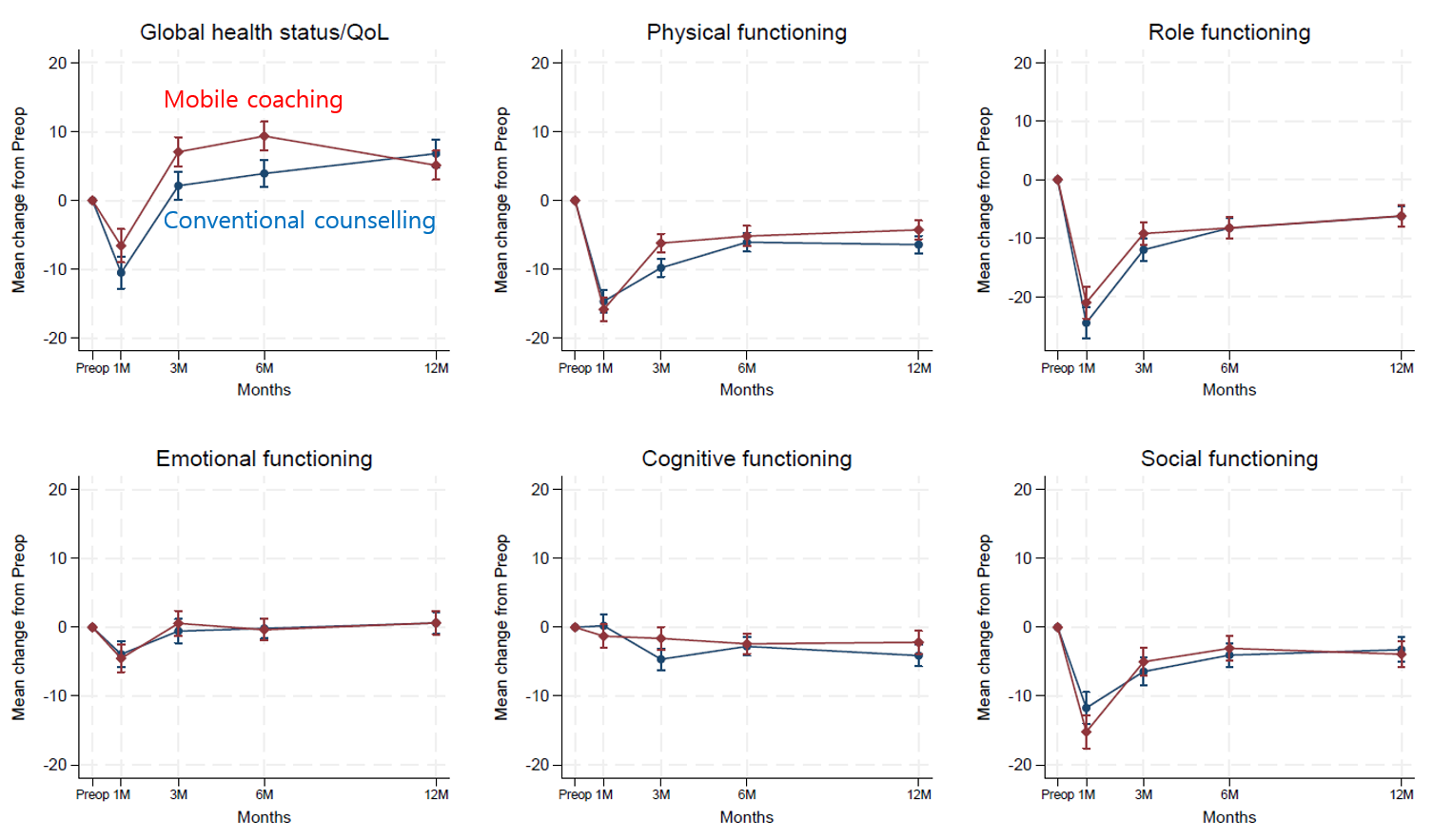

Supplement: Multimedia Appendix 1 [file mhealth_v13i1e75445_app1.png]

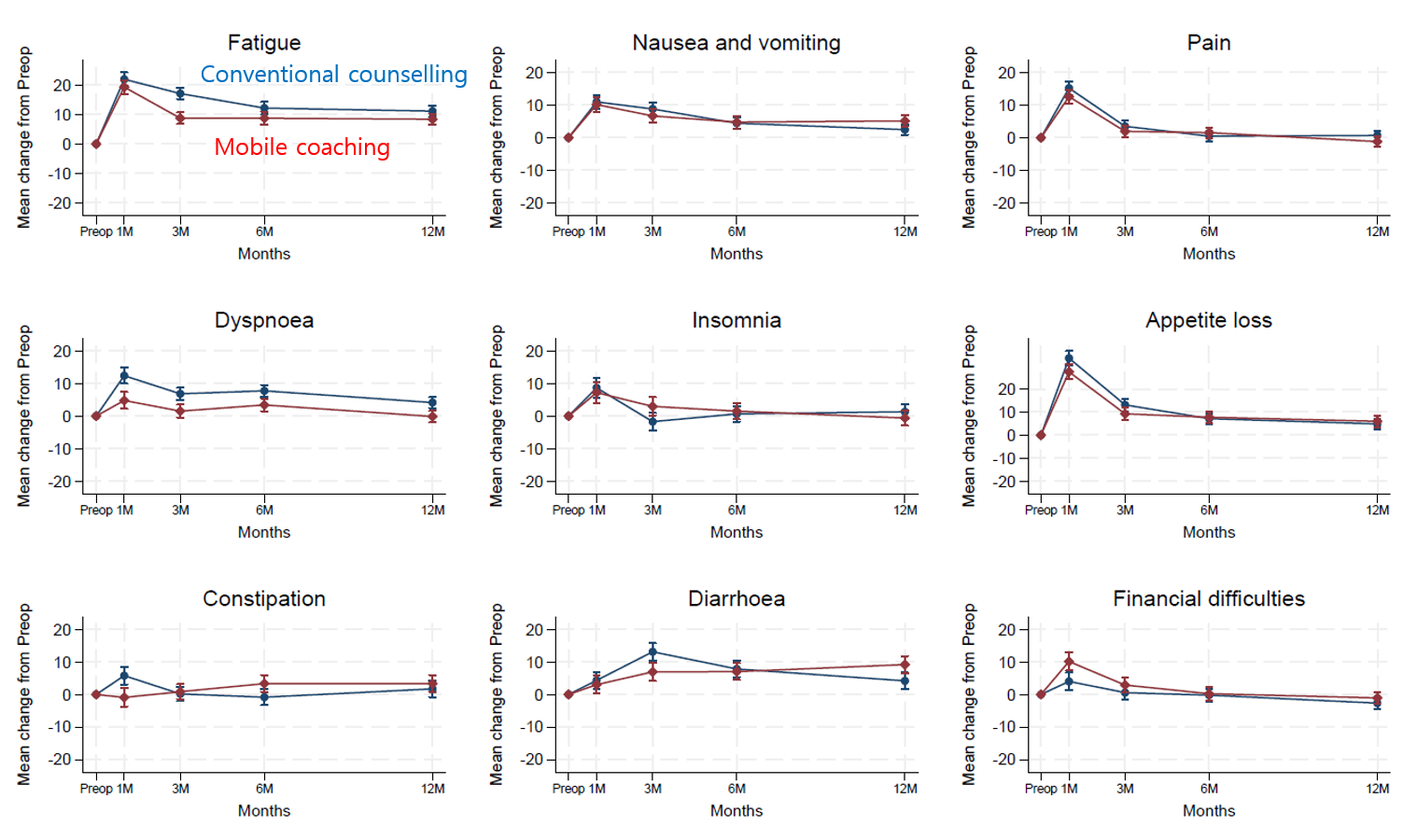

Supplement: Multimedia Appendix 2 [file mhealth_v13i1e75445_app2.png]

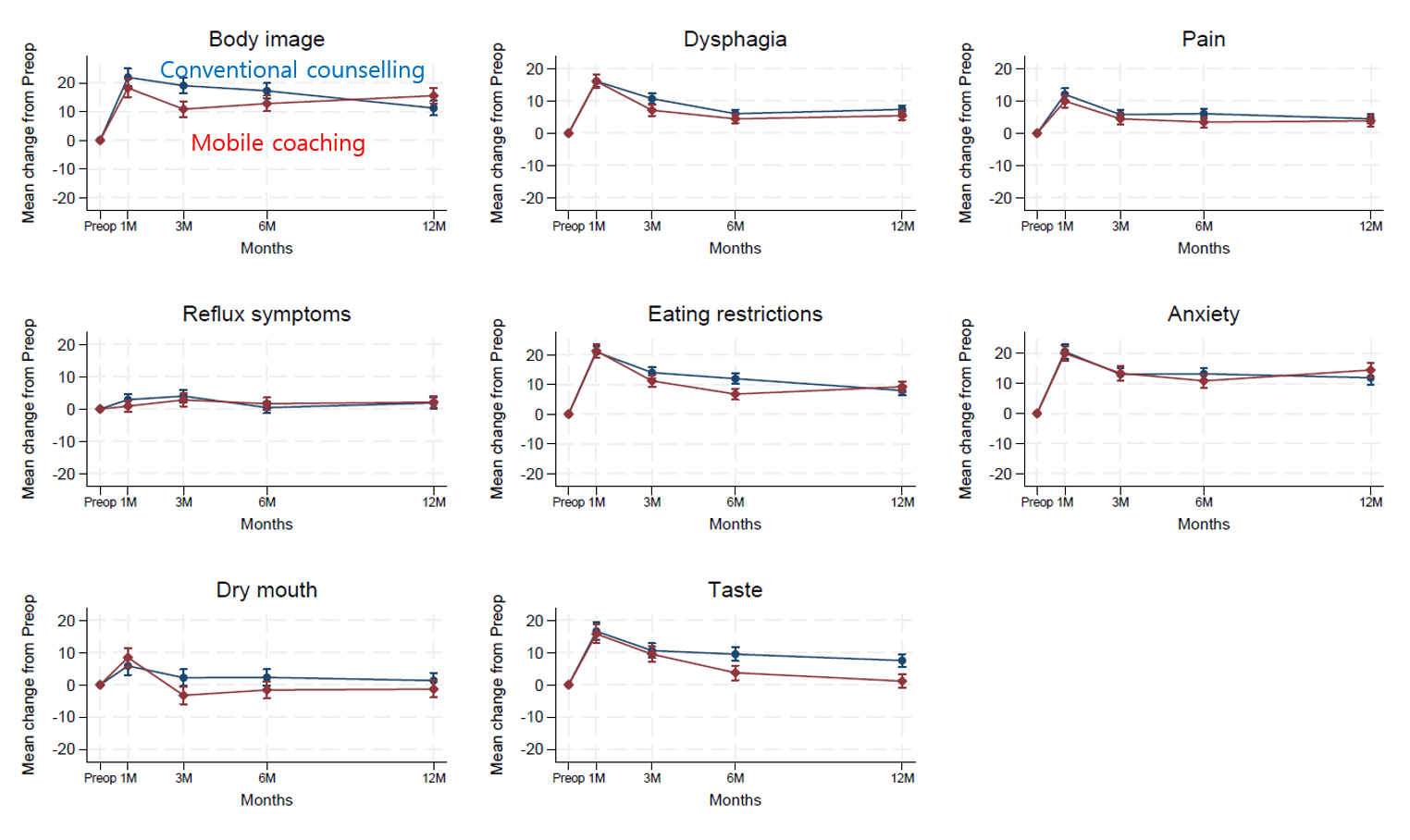

Supplement: Multimedia Appendix 3 [file mhealth_v13i1e75445_app3.png]

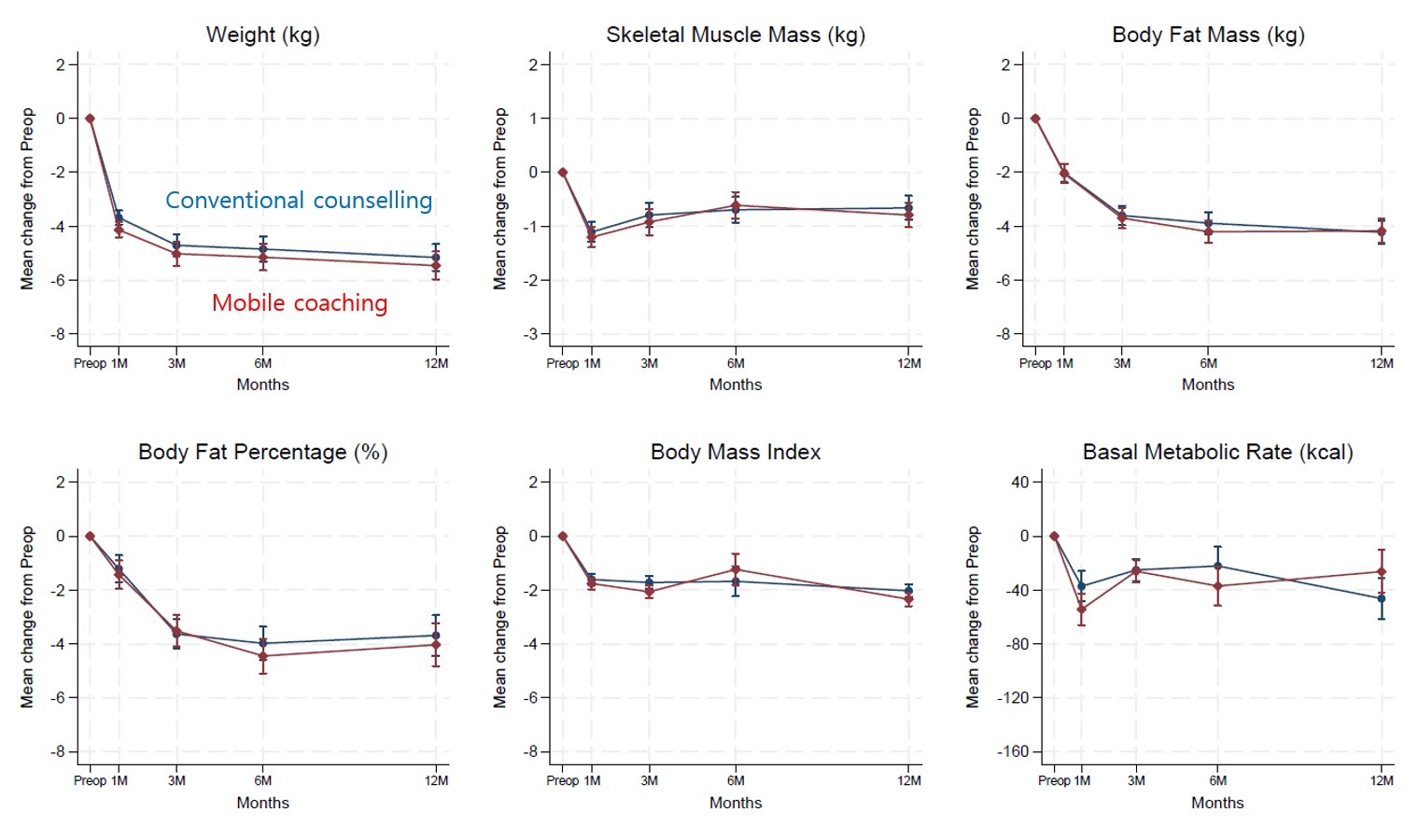

Supplement: Multimedia Appendix 5 [file mhealth_v13i1e75445_app5.png]

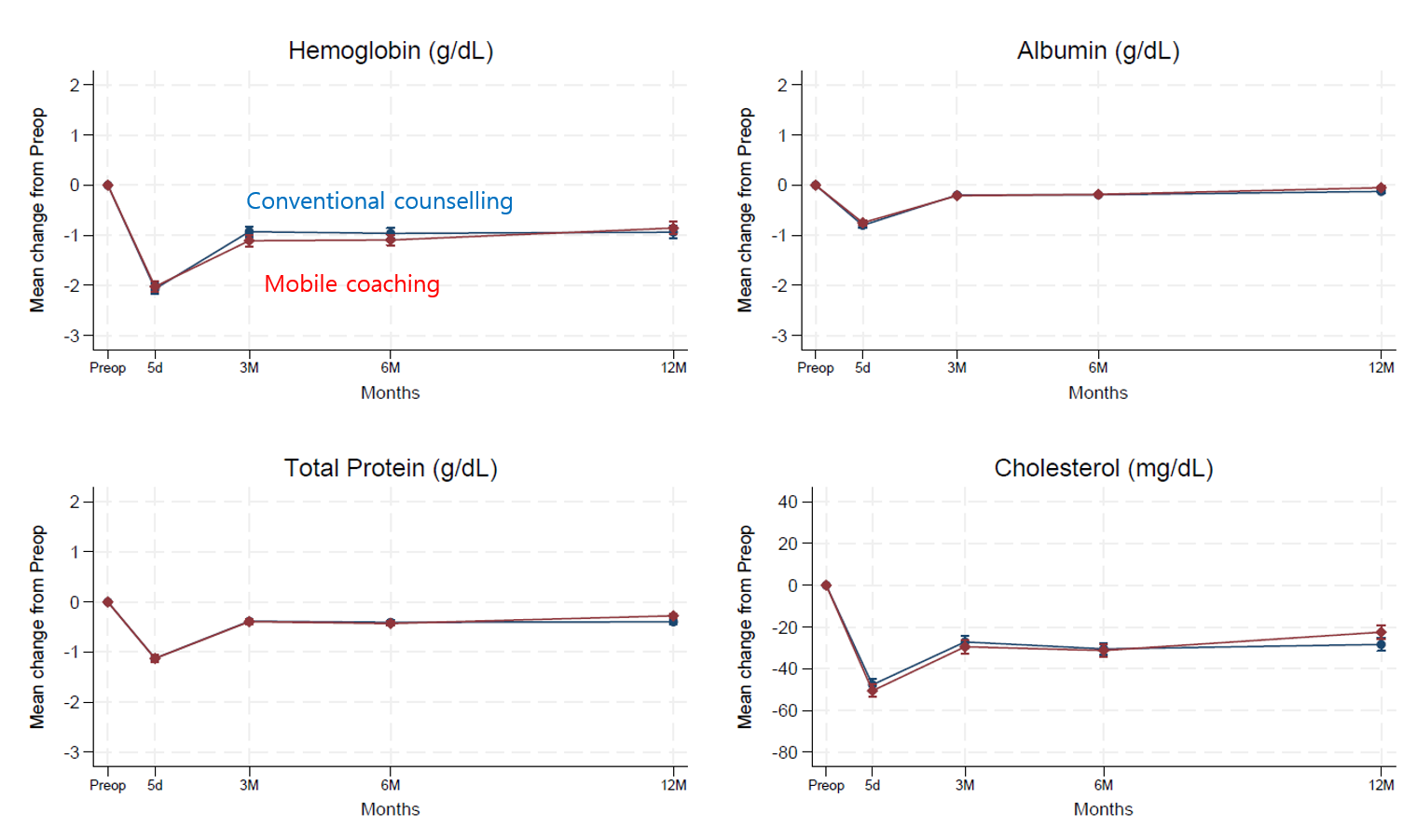

Supplement: Multimedia Appendix 6 [file mhealth_v13i1e75445_app6.png]

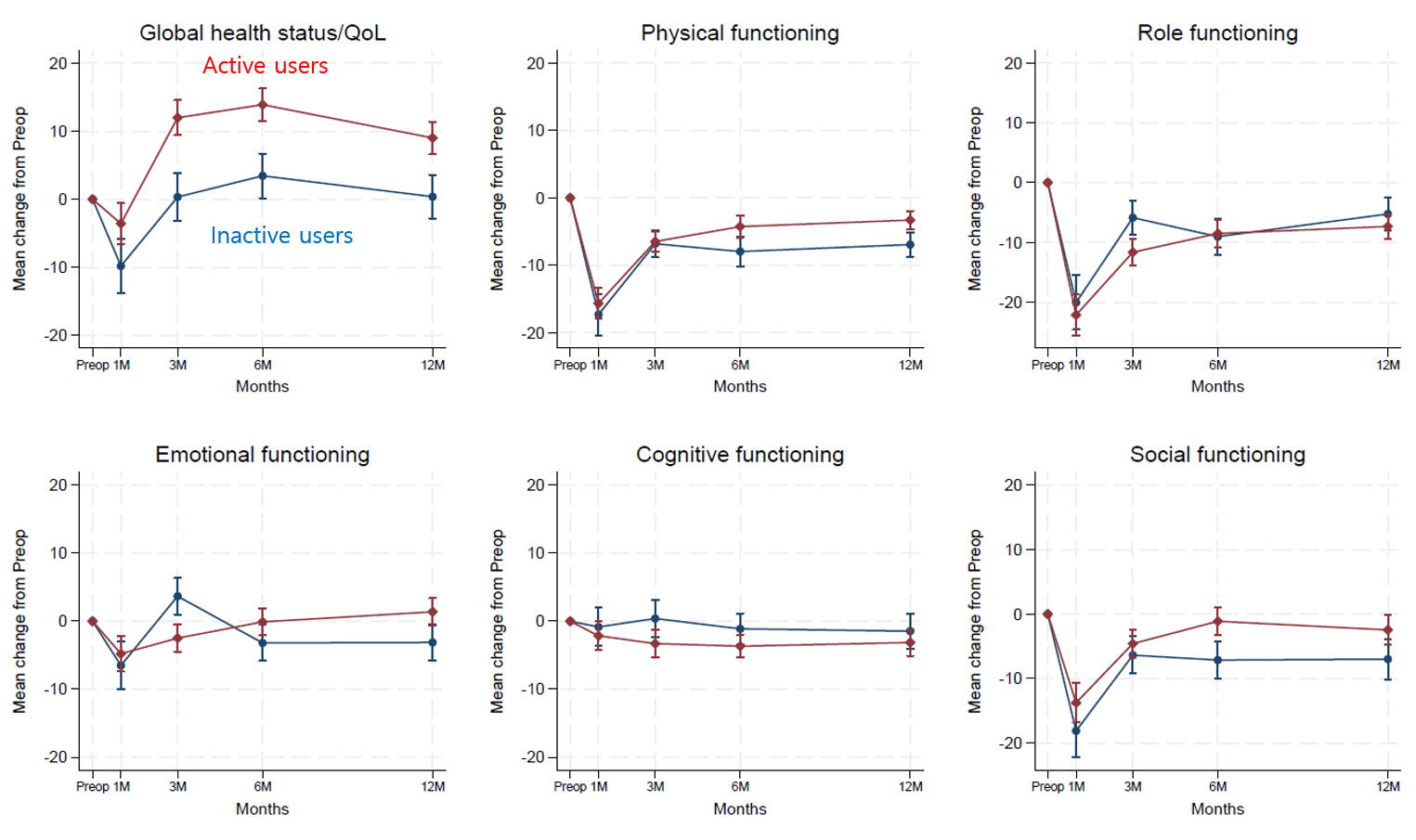

Supplement: Multimedia Appendix 7 [file mhealth_v13i1e75445_app7.png]

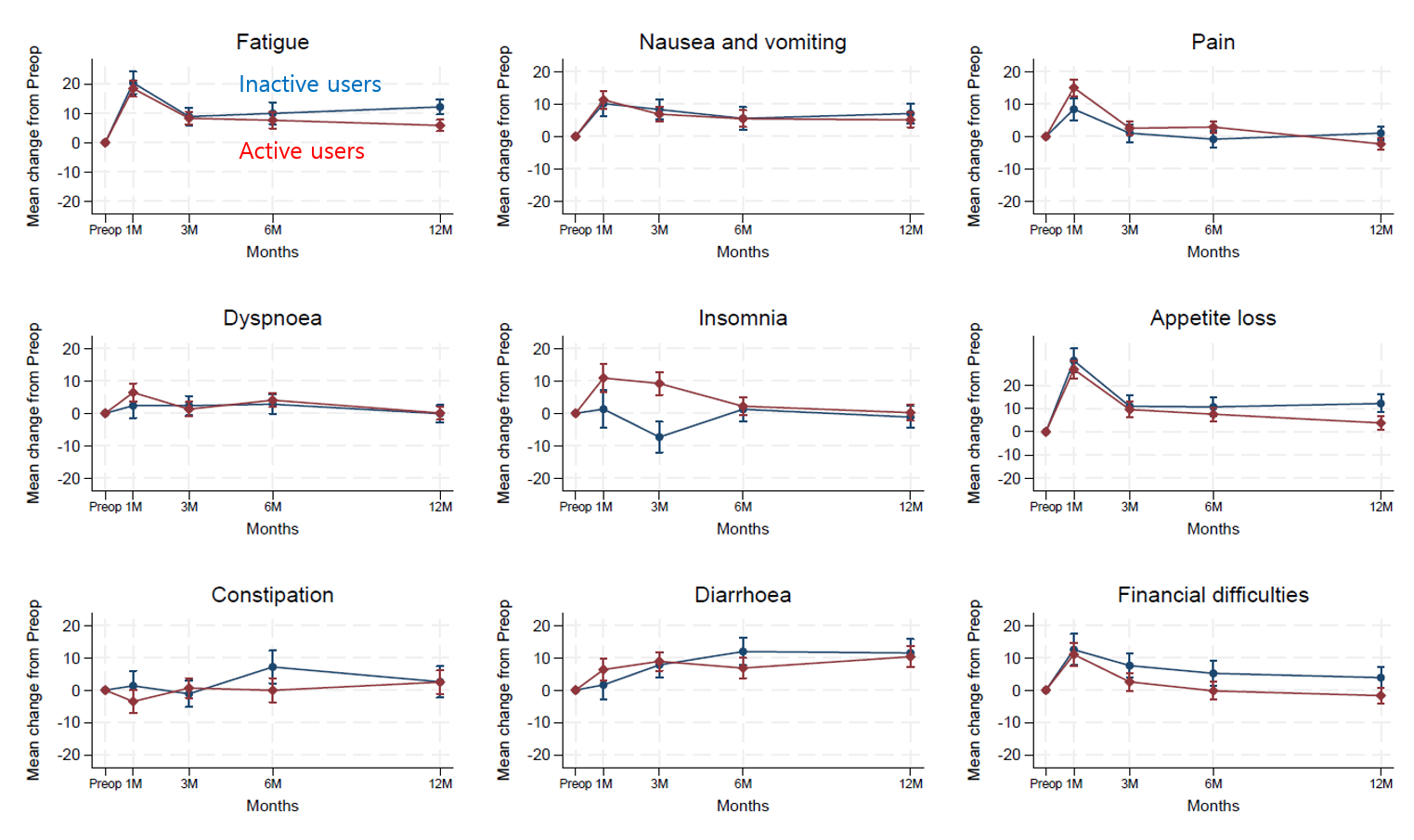

Supplement: Multimedia Appendix 8 [file mhealth_v13i1e75445_app8.png]

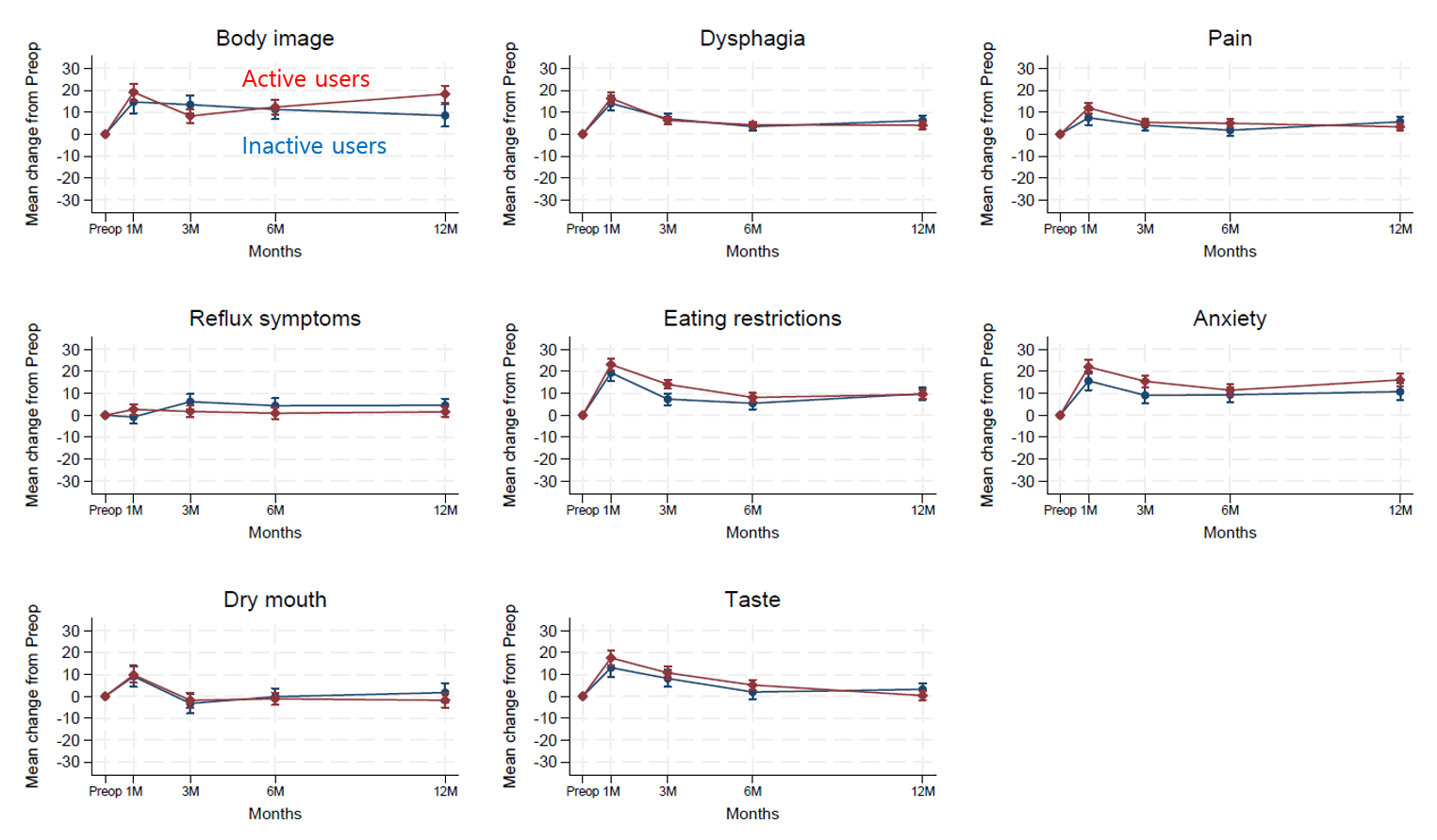

Supplement: Multimedia Appendix 9 [file mhealth_v13i1e75445_app9.png]
