# Supplementary material for: Improvement in Quality of Life After Early Interactive Human Coaching via a Mobile App in Postgastrectomy Patients With Gastric Cancer: Prospective Randomized Controlled Trial
Source: JMIR Mhealth Uhealth. 2025 Dec 18;13:e75445. doi: 10.2196/75445 (PMC12757711; doi:10.2196/75445)
Supplement: Multimedia Appendix 4 [file mhealth_v13i1e75445_app4.docx]

Supplementary Table 1. Subgroup analyses based on age. P values indicate the significance of differences in postoperative QoL between the mobile coaching and conventional counselling groups.

|  | Subscales | Age < 60 years  (n=82) | Age ≥ 60 years  (n=78) |
| --- | --- | --- | --- |
|  |  | *P* value | *P* value |
| EORTC QLQ-C30 | Global health status | .188 | .392 |
|  | Physical functioning | .192 | .836 |
|  | Role functioning | .499 | .613 |
|  | Emotional functioning | .257 | .269 |
|  | Cognitive functioning | .495 | .781 |
|  | Social functioning | .553 | .355 |
|  | **Fatigue** | **.013** | .564 |
|  | Nausea and vomiting | .497 | .417 |
|  | Pain | .616 | .620 |
|  | **Dyspnea** | **.004** | .568 |
|  | Insomnia | .188 | .300 |
|  | Appetite loss | .227 | .886 |
|  | Constipation | .784 | .711 |
|  | Diarrhoea | .733 | .966 |
|  | Financial difficulties | .603 | .291 |
| EORTC QLQ-STO22 | Dysphagia | .332 | .488 |
|  | Pain | .113 | .622 |
|  | Reflux symptoms | .192 | .448 |
|  | Eating restrictions | .136 | .570 |
|  | Anxiety | .767 | .777 |
|  | Dry mouth | .311 | .617 |
|  | Taste | .083 | .590 |
|  | **Body image** | **.017** | .556 |

EORTC QLQ, European Organization for Research and Treatment of Cancer Quality of Life Questionnaire

Supplementary Table 2. Subgroup analyses based on sex. P values indicate the significance of differences in postoperative QoL between the mobile coaching and conventional counselling groups.

|  | Subscales | Male  (n=96) | Female  (n=64) |
| --- | --- | --- | --- |
|  |  | *P* value | *P* value |
| EORTC QLQ-C30 | Global health status | .752 | .131 |
|  | Physical functioning | .811 | .197 |
|  | Role functioning | .988 | .347 |
|  | **Emotional functioning** | .018 | **.027** |
|  | Cognitive functioning | .650 | .889 |
|  | Social functioning | .174 | .161 |
|  | Fatigue | .296 | .059 |
|  | Nausea and vomiting | .781 | .613 |
|  | **Pain** | .334 | **.040** |
|  | Dyspnea | .094 | .078 |
|  | Insomnia | .670 | .775 |
|  | Appetite loss | .665 | .273 |
|  | Constipation | .603 | .302 |
|  | Diarrhoea | .845 | .604 |
|  | Financial difficulties | .048 | .148 |
| EORTC QLQ-STO22 | **Dysphagia** | .658 | **.043** |
|  | **Pain** | .076 | **.037** |
|  | Reflux symptoms | .914 | .685 |
|  | Eating restrictions | .662 | .099 |
|  | Anxiety | .818 | .815 |
|  | **Dry mouth** | .280 | **.019** |
|  | Taste | .493 | .063 |
|  | Body image | .578 | .192 |

EORTC QLQ, European Organization for Research and Treatment of Cancer Quality of Life Questionnaire

Supplementary Table 3. Subgroup analyses based on sex. P values indicate the significance of differences in postoperative QoL between the mobile coaching and conventional counselling groups.

|  | Subscales | Distal gastrectomy  (n=145) | Total gastrectomy  (n=15) |
| --- | --- | --- | --- |
|  |  | *P* value* | *P* value* |
| EORTC QLQ-C30 | Global health status | .164 | .955 |
|  | Physical functioning | .227 | .337 |
|  | Role functioning | .321 | .481 |
|  | Emotional functioning | .876 | .772 |
|  | Cognitive functioning | .475 | .812 |
|  | Social functioning | .886 | .878 |
|  | **Fatigue** | **.022** | .536 |
|  | Nausea and vomiting | .804 | .720 |
|  | Pain | .382 | .753 |
|  | **Dyspnea** | **.001** | .124 |
|  | Insomnia | .949 | .199 |
|  | Appetite loss | .399 | .794 |
|  | Constipation | .809 | .491 |
|  | Diarrhoea | .637 | .717 |
|  | Financial difficulties | .434 | .285 |
| EORTC QLQ-STO22 | Dysphagia | .161 | .857 |
|  | Pain | .307 | .835 |
|  | Reflux symptoms | .530 | .114 |
|  | Eating restrictions | .463 | .895 |
|  | Anxiety | .668 | .144 |
|  | Dry mouth | .258 | .948 |
|  | Taste | .118 | .967 |
|  | Body image | .129 | .510 |

*P-values were calculated using linear mixed models with repeated measures.

EORTC QLQ, European Organization for Research and Treatment of Cancer Quality of Life Questionnaire
