# Supplementary material for: Improvement in Quality of Life After Early Interactive Human Coaching via a Mobile App in Postgastrectomy Patients With Gastric Cancer: Prospective Randomized Controlled Trial
Source: JMIR Mhealth Uhealth. 2025 Dec 18;13:e75445. doi: 10.2196/75445 (PMC12757711; doi:10.2196/75445)
Supplement: Multimedia Appendix 10 [file mhealth_v13i1e75445_app10.docx]

Supplementary Table 1. Correlation between app activity and EROTC QLQ-C30 score.

| EORTC QLQ-C30 | Meal input | Exercise input | Sending messages | Reading articles | Weight input |
| --- | --- | --- | --- | --- | --- |
| Global health status/QoL | 0.292 | 0.185 | 0.366 | 0.200 | 0.065 |
| Physical functioning | 0.133 | 0.117 | -0.009 | 0.143 | 0.091 |
| Role functioning | 0.003 | 0.034 | -0.106 | 0.023 | 0.005 |
| Emotional functioning | 0.011 | 0.130 | 0.026 | 0.092 | -0.060 |
| Cognitive functioning | -0.261 | 0.030 | -0.184 | -0.211 | -0.170 |
| Social functioning | 0.125 | 0.088 | 0.045 | 0.206 | 0.121 |
| Fatigue | -0.114 | -0.101 | -0.095 | -0.167 | -0.017 |
| Nausea and vomiting | -0.228 | -0.063 | -0.175 | -0.196 | -0.129 |
| Pain | 0.031 | 0.045 | 0.005 | -0.100 | 0.004 |
| Dyspnea | -0.127 | -0.103 | 0.034 | -0.077 | -0.109 |
| Insomnia | 0.146 | 0.075 | 0.227 | 0.114 | 0.177 |
| Appetite loss | -0.135 | -0.143 | -0.061 | -0.148 | -0.052 |
| Constipation | 0.037 | 0.006 | -0.109 | -0.004 | 0.071 |
| Diarrhea | -0.100 | 0.280 | -0.054 | -0.246 | -0.244 |
| Financial difficulties | -0.164 | -0.071 | -0.202 | -0.227 | -0.171 |

EORTC QLQ, European Organization for Research and Treatment of Cancer Quality of Life Questionnaire

Supplementary Table 2. Correlation between app activity and EROTC QLQ-STO22 score.

| EORTC QLQ-STO22 | Meal input | Exercise input | Sending messages | Reading article | Weight input |
| --- | --- | --- | --- | --- | --- |
| Body image | -0.125 | -0.088 | -0.109 | -0.186 | -0.101 |
| Dysphagia | -0.061 | -0.226 | -0.190 | -0.099 | -0.024 |
| Pain | 0.002 | -0.128 | 0.021 | -0.115 | -0.038 |
| Reflux symptoms | -0.216 | -0.171 | -0.142 | -0.145 | -0.190 |
| Eating restrictions | 0.063 | -0.106 | 0.109 | 0.077 | 0.046 |
| Anxiety | 0.181 | -0.064 | 0.059 | 0.122 | 0.133 |
| Dry mouth | 0.109 | -0.064 | 0.087 | -0.002 | 0.018 |
| Taste | -0.108 | -0.203 | -0.271 | -0.097 | 0.023 |

EORTC QLQ, European Organization for Research and Treatment of Cancer Quality of Life Questionnaire
